# Pilot Room-Level Acoustic and Physiological Monitoring of Respiratory Disturbance in Pigs Following Experimental *Klebsiella pneumoniae* Challenge

**DOI:** 10.3390/vetsci13060550

**Published:** 2026-06-03

**Authors:** Md Sharifuzzaman, Hong-Seok Mun, Eddiemar B. Lagua, Md Kamrul Hasan, Ahsan Mehtab, Jin-Gu Kang, Hae-Rang Park, Young-Hwa Kim, Chul-Ju Yang

**Affiliations:** 1 Animal Nutrition and Feed Science Laboratory, Department of Animal Science and Technology, Sunchon National University, Suncheon 57922, Republic of Korea; baushossain@gmail.com (M.S.);; 2 Department of Animal Science and Veterinary Medicine, Gopalganj Science and Technology University, Gopalganj 8105, Bangladesh; 3 Department of Multimedia Engineering, Sunchon National University, Suncheon 57922, Republic of Korea; 4 Interdisciplinary Program in IT-Bio Convergence System (BK21 Plus), Sunchon National University, Suncheon 57922, Republic of Korea; 5 Department of Poultry Science, Sylhet Agricultural University, Sylhet 3100, Bangladesh; 6 Interdisciplinary Program in IT-Bio Convergence System (BK21 Plus), Chonnam National University, Gwangju 61186, Republic of Korea

**Keywords:** audio spectrogram transformer, *Klebsiella pneumoniae*, artificial intelligence, precision livestock farming, bioacoustics, cough detection, swine

## Abstract

Respiratory disease remains a major challenge in pig production. This two-room pilot study evaluated whether room-level acoustic monitoring combined with physiological measurements could provide an early warning after an experimental *Klebsiella pneumoniae* challenge. Forty growing pigs balanced by sex and body weight were housed for 28 days in one control room and one challenged room (20 pigs/room; four pens/room). Challenged pigs were intranasally inoculated on days 8, 12, 16, and 20 with a culture whose dose was retrospectively verified by serial-dilution plating. Nasal and fecal samples were cultured on Klebsiella ChromoSelect agar, and colonies with expected morphology were enumerated as presumptive *Klebsiella/K. pneumoniae* colonies. A fine-tuned Audio Spectrogram Transformer (AST) classified five sound classes from facility-specific audio and was evaluated by group-blocked hold-out testing, five-fold group-blocked cross-validation, temporal deployment validation, and window-threshold sensitivity analysis. The model achieved hold-out macro-F1 of 0.947, five-fold macro-F1 of 0.928 ± 0.019, and 24 h deployment macro-F1 of 0.914. Presumptive nasal bacterial load was higher in challenged pigs at 1-week post-inoculation (log_10_ 4.03 vs. 0.67). Group-size-standardized cough detections were also higher in the challenged room (54.84 vs. 36.80 detections/day), and daily coughing first exceeded the baseline threshold on day 8. Thresholds of 0.764 (control) and 1.115 (treatment) were obtained from an integrated score that included coughing, sneezing, ear temperatures, rectal temperature, and respiration rate; the treatment score and treatment–control contrast score first surpassed the threshold on day 8, and daily multimodal scores varied between groups (t = −6.636, *p* < 0.001). Integrated score improved discrimination of post-inoculation disturbance compared with cough detections alone (leave-one-day-out AUROC: 0.94 vs. 0.88). Because each condition was represented by one room, findings are exploratory temporal contrasts, not replicated treatment effects or a stand-alone diagnostic test.

## Simple Summary

Respiratory problems in pigs can reduce growth, welfare, and farm profitability, but early warning signs are often missed during routine checks. In this two-room pilot study, 40 growing pigs were monitored for 28 days after repeated nasal exposure of one group to *Klebsiella pneumoniae*. Continuous room-level sound recording was analyzed with an artificial intelligence model to detect coughing, sneezing, vocalizations, and silence. These acoustic outputs were interpreted together with bacterial recovery, body temperature, breathing rate, blood variables, fecal score, and growth performance. The challenged room showed higher nasal bacterial recovery, poorer growth, higher coughing activity, and more coughing risk windows after inoculation. A combined acoustic–physiological score also separated the challenged and control rooms from day 8 onward. These findings suggest that continuous room-level sound monitoring, especially when combined with simple physiological measurements, may support early warning of respiratory disturbance under controlled challenge conditions. Future studies should validate this approach in replicated rooms and commercial farms before routine use.

## 1. Introduction

Respiratory diseases remain one of the most important health challenges in global swine production, causing reduced growth performance, increased mortality, and substantial economic losses. Major pathogens associated with respiratory disease in pigs include porcine reproductive and respiratory syndrome virus (PRRSV), *Mycoplasma hyopneumoniae*, and several opportunistic bacterial agents [1,2,3]. These infections impair average daily gain (ADG), reduce feed efficiency, and delay pigs from reaching market weight, ultimately lowering farm profitability [4,5,6]. In addition, respiratory diseases compromise animal welfare by inducing clinical signs such as coughing, fever, lethargy, and respiratory distress [7,8].

Among opportunistic pathogens, *Klebsiella pneumoniae* has increasingly been recognized as a respiratory and systemic pathogen of veterinary importance in pigs. Recent reports from pig farms and diagnostic investigations have described *K. pneumoniae* carriage, virulence determinants, antimicrobial resistance, and links with pneumonia, septicemia, and respiratory lesions [9,10]. The bacterium can colonize the upper respiratory tract and has been isolated from nasal cavities and respiratory tissues associated with pneumonia and lung lesions [11]. Its pathogenicity is linked to several virulence mechanisms, including capsule polysaccharide production, siderophore-mediated iron acquisition, and biofilm formation, which enhance bacterial survival and immune evasion within the host [11,12]. Infected pigs may therefore develop respiratory distress and systemic infections, particularly under conditions of stress or compromised immunity [11,13]. These characteristics make *K. pneumoniae* a relevant model organism for evaluating early monitoring approaches in controlled pig challenge studies.

Early detection of respiratory disease is essential for effective herd health management. Several physiological indicators have been proposed for monitoring respiratory health in pigs, including respiration rate and body temperature [14,15]. Non-invasive thermal measurements such as ear-base temperature have also been suggested as indicators of physiological stress and disease [15]. In addition, coughing frequency and behavioral changes are widely used as indicators of respiratory disturbance in pigs [16,17].

Recent advances in bioacoustics and artificial intelligence (AI) have created new opportunities for automated respiratory disease detection. Machine learning models, particularly deep learning approaches, have shown high accuracy in identifying respiratory conditions through analysis of cough and breathing sounds [18,19,20,21]. However, most monitoring systems rely on a single type of signal, such as coughing activity or physiological changes. Because respiratory infections often trigger complex physiological and acoustic responses, integrating multiple indicators may provide a more reliable framework for early disease detection.

Therefore, this pilot study investigated whether a room-level sound-classification system, interpreted together with physiological and bacteriological data, could indicate respiratory disturbance after an experimental *K. pneumoniae* challenge. The specific objectives were to: (i) verify nasal and fecal *K. pneumoniae* recovery after challenge; (ii) describe growth, physiological, biochemical, and fecal responses; (iii) validate a facility-specific Audio Spectrogram Transformer (AST) model for five-class pig sound classification using leakage-aware and temporal checks; (iv) quantify room-level coughing and related acoustic patterns after challenge; and (v) test whether a baseline-standardized acoustic–physiological score improved early-warning performance relative to cough detections alone. Because this study used one room per condition, the analyses were designed as exploratory temporal contrasts within a two-room pilot setting.

## 2. Materials and Methods

### 2.1. Animals, Experimental Design and Bacterial Challenge

**Figure 1:**
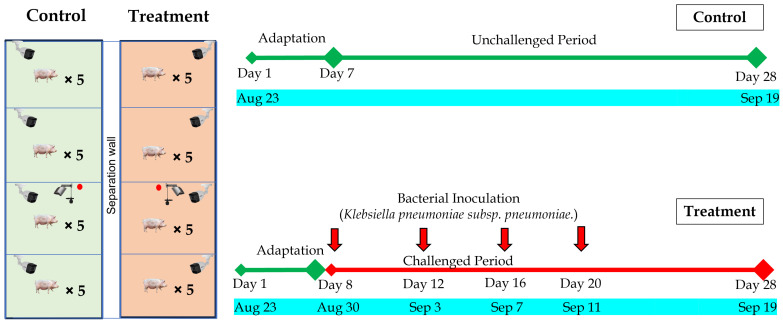
Experimental design and monitoring setup for the *Klebsiella pneumoniae* challenge study. The left panel shows the housing layout for the control and treatment groups, pen arrangement, and placement of cameras, the room-level microphone, and the environmental sensor (red dot). The right panel shows the 28-day experimental timeline, including the 7-day adaptation period and intranasal inoculation days.

The experiment was conducted from 23 August to 19 September 2025 at the experimental pig facility of Sunchon National University. Forty clinically healthy growing pigs (20 males and 20 females) of the [(Large White × Landrace) × Duroc] crossbred lineage were used. Inclusion criteria were normal appetite, absence of visible respiratory signs, no antibiotic treatment during the previous 14 days, and body weight within the target grower range. Pigs showing fever, persistent coughing, nasal discharge, diarrhea, severe lameness, or other clinical abnormalities before allocation were to be excluded; no pig met these exclusion criteria at enrollment. Pigs were allocated by sex- and body-weight-stratified randomization to a control room (n = 20) or a challenged room (n = 20). Each room contained four pens with five pigs per pen (2.35 m × 2.90 m). The two rooms were managed identically except for the inoculation procedure, but each condition was represented by one room only. Acoustic monitoring was performed with one centrally positioned microphone per room (Figure 1). Accordingly, acoustic, environmental, and integrated-score analyses are interpreted as room-level temporal contrasts rather than replicated treatment effects.

Automatic wet-dry feeders (LFS-120, ION-TECH Co., Ltd., Seoul, Republic of Korea) were used to give animals unlimited access to a commercial grower food while maintaining a conventional lighting schedule (16 h light and 8 h dark). A farm monitoring system (Farm Note, NareTrends Inc., Bucheon, Republic of Korea) was used to track environmental parameters (NH_3_, CO_2_, temperature, and relative humidity) at five-minute intervals. These procedures followed routine husbandry and environmental-monitoring approaches used in pig respiratory health studies [7,8,14,15].

This study comprised a bacterial challenge phase after a 7-day adaptation period (days 1–7). On days 8, 12, 16, and 20, pigs in the treatment room received an intranasal injection of a suspension of *Klebsiella pneumoniae* (KCTC 12385) from the Korean Collection for Type Cultures. Control pigs received sterile saline by the same route and at the same handling times to maintain comparable procedural exposure.

The bacterial culture was grown on nutrient agar at 37 °C for 24 h and suspended in sterile saline to prepare a turbid inoculum. At the time of dosing, the inoculum was standardized visually rather than by optical density or pre-dose plate counting. To estimate the likely viable concentration produced by this preparation procedure, the inoculum-preparation protocol was subsequently repeated under the same culture conditions and quantified by serial-dilution plating. This verification indicated an approximate concentration of 1.1 × 10^8^ CFU/mL. Each challenged pig received 10 mL of inoculum (5 mL per nostril), corresponding to approximately 1.1 × 10^9^ CFU per pig, and control pigs received 10 mL of sterile saline by the same route. Because the administered dose was not plate-counted before dosing, the concentration should be interpreted as an estimated protocol-based dose rather than a prospectively confirmed dose.

### 2.2. Bacterial Sampling and Enumeration

Nasal swabs and fecal samples were collected before the first inoculation (0HBI) and at 1, 2, and 3 weeks after the first inoculation, following standard respiratory-sampling and microbiological procedures [22,23,24]. Swabs were vortexed in 10 mL sterile saline, and fecal samples (1 g) were homogenized in 9 mL saline (1:10, *w*/*v*). From each suspension, 20 µL (0.02 mL) was plated onto Klebsiella ChromoSelect agar and incubated at 37 °C for 18–24 h. According to the manufacturer’s specification, this medium is designed for selective isolation and enumeration of *Klebsiella* species, with *K. pneumoniae* producing purple–magenta mucoid colonies, while several common Gram-negative fecal contaminants are inhibited. Therefore, colonies showing the expected purple–magenta mucoid morphology were manually enumerated as presumptive *Klebsiella/K. pneumoniae* colonies. Bacterial load was expressed as CFU per swab or per gram of feces after correction for plated volume and dilution. Because no molecular, MALDI-TOF, sequencing, or biochemical confirmation was performed for any recovered colonies, the culture results were interpreted as presumptive recovery of the challenge organism rather than definitive species-level confirmation.

### 2.3. Growth Performance and Physiological Measurements

Body weight and feed intake were recorded weekly. Body weight gain (BWG), average daily gain (ADG), average daily feed intake (ADFI), and feed conversion ratio (FCR) were calculated at the pen level. For overall growth summaries, the pen was treated as the experimental unit (four pens per room), while the lack of room-level replication was explicitly retained as a limitation for treatment inference.

Physiological measurements included body temperature and respiration rate. Body temperature was recorded four times daily (00:00, 06:00, 12:00, and 18:00 h) using three methods: ear surface temperature measured via thermal imaging, inner ear temperature, and rectal temperature. Respiration rate was also measured four times daily (00:00, 06:00, 12:00, and 18:00 h) from video recordings by counting breaths per minute in all pigs. Measurements were averaged by pen and room for descriptive analyses. These measurements were used as contextual physiological indicators and as inputs to the integrated score; they were not treated as independent confirmation of infection on their own.

Blood samples were collected via jugular venipuncture at 0HBI and at 1, 2, and 3-weeks post-first inoculation. Plasma was separated by centrifugation and stored at −20 °C until analysis. Albumin and total protein concentrations were determined using automated biochemical methods at a certified laboratory (Yongin, Republic of Korea). Blood was collected from all pigs within each pen, and the resulting values were averaged to obtain a single pen-level mean. The pen (n = 4 per group) was considered the experimental unit for statistical analysis.

Fecal consistency was scored daily using a standardized 4-point scale (0 = normal, 3 = severe diarrhea).

### 2.4. Acoustic Monitoring and Sound Classification

For acoustic analysis, audio tracks were extracted from archived video files and stored as MP3 files (124 kbps, stereo, 44.1 kHz) with synchronized SMI timestamp files. Five sound classes were defined: coughing, sneezing, aggressive vocalizations, normal vocalizations, and silence. Coughing was defined as a sharp, forceful expiratory sound; sneezing as a brief, explosive nasal sound; aggressive vocalizations as squeals or distress calls associated with social conflict; normal vocalizations as routine grunts, oinks, and snorts; and silence as periods without biologically relevant acoustic activity.

Model development used a facility-specific pig-sound dataset assembled before the present 28-day experiment. The dataset consisted of 8094 manually labeled clips collected from growing pigs housed in the same university facility during separate studies conducted between February and June 2023, covering an approximate body-weight range of 28–80 kg. Only recordings from contemporaneous control animals were included; therefore, the dataset was treated as a site-specific pig-sound library rather than a clinically defined respiratory-disease dataset. The labeled clips included coughing (n = 2995), sneezing (n = 1414), aggressive vocalizations (n = 879), normal vocalizations (n = 1143), and silence (n = 1663).

Audio labeling was performed in Label Studio (v1.12) using synchronized playback and waveform inspection. Annotators were trained using representative examples, and uncertain clips were reviewed by a second trained annotator. Class definitions were checked by an expert veterinarian and an experienced farm manager. To assess annotation consistency, 1600 clips, representing approximately 20% of the dataset, were independently re-labeled by a blinded second annotator. Inter-annotator agreement was substantial, with Cohen’s kappa of 0.82. Discrepant clips were resolved by consensus before final model training. All labeled clips were exported as 16-bit WAV files at 44.1 kHz.

A pretrained Audio Spectrogram Transformer [25,26] (MIT/ast-finetuned-audioset-10-10-0.4593) was fine-tuned for five-class classification. To reduce leakage risk, clips from the same parent recording, recording date, and animal batch were kept within the same data split or fold. The model was evaluated using a group-blocked 70/10/20 hold-out split and five-fold group-blocked cross-validation. Clips were converted to mono, resampled to 16 kHz, and standardized to 1.0 s by trimming or zero-padding. Across 600 manually reviewed cough-like, sneeze, aggressive, normal, and silence windows, most biologically relevant energy was below 7.2 kHz, and no class-defining spectral component was consistently observed above 8 kHz. Resampling to 16 kHz, therefore, retained the relevant 0–8 kHz band while reducing computational cost and matching the AST preprocessing pipeline. Training augmentation included random temporal shift, gain perturbation, and additive barn-noise mixing, whereas validation and test clips were not augmented.

Class imbalance was addressed using inverse-frequency class weights, and the final checkpoint was selected using validation macro-F1 rather than accuracy. The final model used a learning rate of 2 × 10^−5^, batch size of 8, and up to 12 epochs with early stopping. Temporal deployment validation was performed on 24 manually annotated hours. Background-noise matching and room-balanced evaluation were also performed to examine whether predictions were influenced by room- or ventilation-specific acoustic fingerprints. Performance remained stable after excluding high-noise windows and after matching background-noise distributions, with noise-matched macro-F1 values of 0.908–0.921. Because no separate-farm validation was available, all AST performance metrics are interpreted as internal, site-specific validation rather than evidence of external generalizability or direct diagnosis of *K. pneumoniae* infection.

### 2.5. Acoustic Data Processing

For deployment, continuous recordings were segmented into overlapping 1.0 s windows with a 0.6 s hop. Because most annotated coughs, sneezes, and routine vocal events were shorter than 1 s, each window was assigned one dominant class; thus, outputs represent window-level room detections rather than exact individual-pig vocal counts. Windows with maximum class probability < 0.7 were reassigned to silence, synchronized to clock time using SMI files, and summarized as hourly detection counts, acoustic time, and percentage occupancy. Count and duration indices were standardized by the number of pigs per room for descriptive comparison, but were interpreted only as room-level acoustic indices.

To assess robustness, adjacent coughing windows separated by ≤1.2 s were merged into single event episodes, and sensitivity analyses repeated the main comparisons using probability thresholds of 0.60, 0.70, and 0.80 and hop lengths of 0.3, 0.6, and 1.0 s. The post-inoculation increase in treatment-room coughing remained directionally unchanged across settings, with daily coughing trajectories highly concordant with the primary configuration (r = 0.93–0.98). These checks support the robustness of the room-level pattern while confirming that absolute counts are algorithm-dependent.

Hourly sound event counts and durations were summarized for aggressive vocalizations, coughing, normal vocalizations, and sneezing. Daily acoustic variables were obtained by aggregating hourly values. Circadian patterns were examined from the mean hourly coughing profile across the 28-day experiment.

For hourly risk-window analysis, group-specific coughing thresholds were computed from the adaptation period (days 1–7) as μ + 2σ, where μ and σ represent the mean and standard deviation of hourly coughing detections within each group. Hours exceeding the corresponding threshold were classified as coughing risk windows, and their frequencies were summarized by day and by hour of occurrence.

For daily early-warning analysis, daily coughing detections were smoothed using a centered 3-day rolling mean. A baseline threshold was calculated from days 1–7 as μ + 2σ and used to identify the first post-baseline day on which coughing exceeded the expected pre-challenge range.

Manual labeling and model training were performed on Windows 11 workstations equipped with Intel^®^ Core™ processors, 32–64 GB RAM, and NVIDIA RTX-class GPUs (6–24 GB dedicated memory).

### 2.6. Integrated Multimodal Early-Warning Analysis

To evaluate whether combined daily animal-response signals improved respiratory surveillance, an integrated acoustic–physiological early-warning score was constructed from room-level acoustic indices and room-averaged physiological variables. Daily coughing and sneezing detections formed the acoustic component, while ear base temperature (EBT), inner ear temperature (IET), rectal temperature (RT), and respiration rate (RR) formed the physiological component. For each variable, group-specific baseline statistics were calculated from days 1–7 and converted to baseline-standardized anomaly scores. Acoustic and physiological anomaly scores were then averaged with equal weighting to generate a daily integrated score for each group. Equal weighting was used as the primary prespecified rule because the pilot design did not provide an independent training cohort large enough to learn stable weights without overfitting. Sensitivity analyses compared the equal-weight score with acoustic-only, physiological-only, inverse-variance-weighted, and leave-one-day-out logistic-weighted scores. Group-specific thresholds were defined as baseline mean + 2 SD, and a treatment–control contrast score was calculated as the daily difference between the treatment and control integrated scores using the same baseline rule. These fusions, therefore, integrated acoustic and physiological variables only; environmental, bacteriological, and blood variables were retained as contextual layers rather than fused inputs.

### 2.7. Statistical Analysis

Bacterial counts were transformed as log_10_(CFU + 1) prior to analysis. Bacterial and blood variables were summarized at the pen level where appropriate. Growth performance was analyzed using pen-level summaries with linear mixed-effects models including group, week, and group × week; however, because pens were nested within a single room per condition, these results are descriptive room-associated contrasts rather than independent replicated treatment effects.

Blood parameters were analyzed using mixed repeated-measures ANOVA with group and time effects. Acoustic variables were analyzed as repeated room-level time series. *p*-values for acoustic, environmental, and integrated-score outputs are to be seen only as exploratory screening statistics because repeated hourly and daily observations from the same two rooms are not independent biological replicates. Effect direction, temporal timing, baseline-threshold exceedance, and sensitivity analyses were emphasized over confirmatory treatment inference; bootstrap confidence intervals were reported where applicable for selected pen-level summaries.

Statistical analyses were performed using Python (v3.11) with pandas, numpy, scipy, statsmodels, scikit-learn, and matplotlib. Leave-one-day-out validation was used for early-warning sensitivity analysis. Statistical significance was set at *p* < 0.05 for descriptive screening, but conclusions were framed according to the two-room pilot design.

## 3. Results

### 3.1. Performance of the Sound Classification Model

Within the facility-specific hold-out evaluation, the selected AST model achieved a validation macro-F1 of 0.953—the prespecified model-selection criterion—and an independent test macro-F1 of 0.947, with a corresponding test accuracy of 0.949 (Table 1). Five-fold group-blocked cross-validation yielded a mean macro-F1 of 0.928 ± 0.019, and the 24 h temporal deployment validation yielded a macro-F1 of 0.914. These results support internal sound-classification performance, but they do not demonstrate cross-farm generalization or infection diagnosis.

Operational validation showed strong agreement between automated and manual hourly counts for aggressive sounds (r = 0.976), coughing (r = 0.998), normal vocalizations (r = 0.995), and sneezing (r = 0.993). After event consolidation and threshold-sensitivity testing, treatment-room coughing trajectories remained directionally stable across windowing settings. These results support the temporal consistency of the room-level deployment pipeline within this dataset; however, the validation subset represented only 24 h of observation, therefore providing limited evidence about long-duration error accumulation or external robustness.

**Figure 2:**
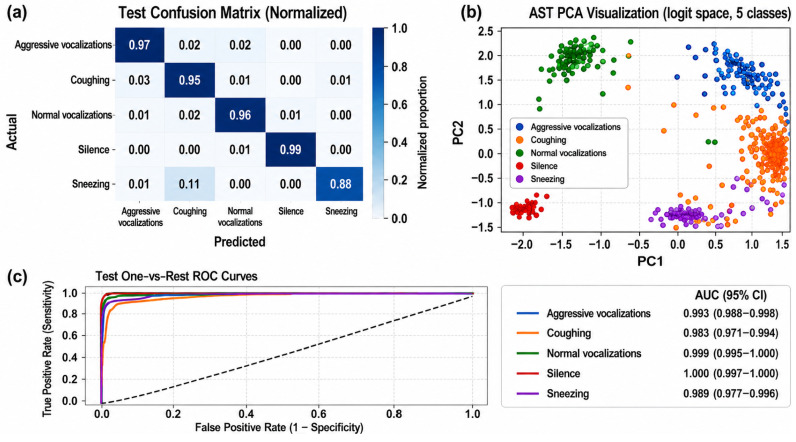
Performance of the fine-tuned Audio Spectrogram Transformer (AST) model for multiclass pig sound classification. (**a**) Normalized confusion matrix for the five sound classes. (**b**) Principal component analysis (PCA) of model output logits showing separation of sound classes in the learned feature space. (**c**) Receiver operating characteristic (ROC) curves demonstrating strong class-wise discrimination performance on the independent test set, the dashed diagonal line represents chance-level classification.

The normalized confusion matrix (Figure 2a) indicates minimal misclassification between classes, while PCA visualization (Figure 2b) demonstrates separation of sound categories in the learned feature space. Furthermore, ROC analysis (Figure 2c) shows high AUC values for all classes, confirming the performance and feasibility of the proposed multiclass acoustic detection framework within the present facility.

### 3.2. Presumptive Recovery of Klebsiella/K. pneumoniae from Nasal and Fecal Samples

**Figure A1:**
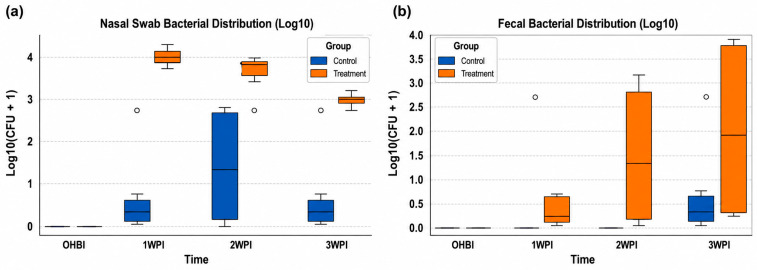
Temporal dynamics of *Klebsiella pneumoniae* detection in biological samples after an experimental challenge. (**a**) Nasal swab bacterial load in control and treatment pigs at 0HBI, 1WPI, 2WPI, and 3WPI. (**b**) Fecal bacterial load at the same sampling points. Values are expressed as mean ± SD log_10_ CFU per swab for nasal samples and per gram of feces for fecal samples, showing predominant respiratory colonization with limited fecal shedding.

In the nasal swab samples, no purple–magenta mucoid colonies consistent with presumptive *Klebsiella/K. pneumoniae* were detected at 0HBI in either group, indicating the absence of detectable culturable presumptive target colonies before inoculation (Table 2 and Figure A1a). Following inoculation, the treatment group exhibited markedly higher presumptive nasal *Klebsiella/K. pneumoniae* counts than the control group. At 1-week post-first inoculation (1WPI), mean presumptive bacterial counts reached log_10_ 4.03 in the treatment group compared with log_10_ 0.67 in controls, and this difference was statistically significant according to both Welch’s *t*-test and the Mann–Whitney U test. At 2WPI, presumptive colony counts declined in the treatment group but remained numerically higher than in controls, with differences approaching statistical significance. By 3WPI, presumptive nasal counts remained significantly higher in the treatment group, supporting persistent recovery of the challenge-associated organism from the nasal cavity. Cumulative presumptive nasal bacterial burden assessed using area under the curve (AUC) was significantly greater in treatment pigs than in controls.

Fecal presumptive *Klebsiella/K. pneumoniae* recovery remained minimal during the early sampling periods in both groups (Table 2 and Figure A1b). Although the treatment group exhibited numerically higher fecal presumptive colony counts at 2WPI and 3WPI, these differences were not statistically significant at any sampling point. Similarly, cumulative fecal presumptive bacterial burden (AUC) did not differ significantly between groups. Overall, these findings suggest that experimental challenge was associated mainly with presumptive respiratory/nasal colonization, with limited evidence of gastrointestinal shedding. Because recovered colonies were identified based on selective chromogenic agar morphology only, these results should be interpreted as presumptive recovery of *Klebsiella/K. pneumoniae*-like colonies rather than definitive species-level confirmation of *K. pneumoniae*.

### 3.3. Growth Performance

**Figure A2:**
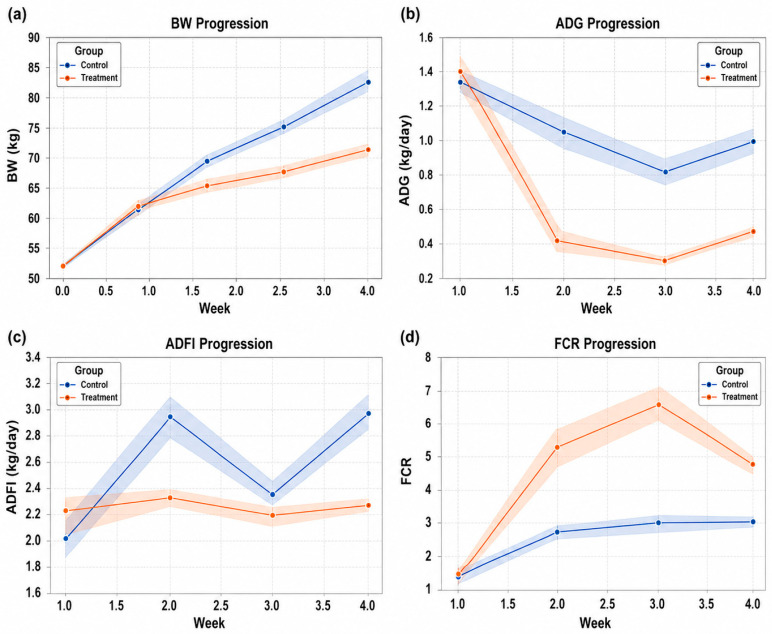
Weekly growth performance trajectories of control and *Klebsiella pneumoniae*-challenged pigs during the 28-day experiment. Panels show changes in (**a**) BW: body weight, (**b**) ADG: average daily gain, (**c**) ADFI: average daily feed intake, and (**d**) FCR: feed conversion ratio, illustrating the progressive reduction in growth performance in treatment pigs after inoculation.

Body weight increased significantly over time during the experimental period (descriptive week effect: *p* < 0.001). The challenged room showed a lower growth trajectory after inoculation, and the group × week term suggested progressive divergence between rooms (Table 3 and Figure A2). Because pigs and pens were nested within one room per condition, these outputs should be read as pen-level summaries within a two-room challenge setting rather than as independently replicated treatment effects.

Average daily gain (ADG) showed a significant decline over time. While no overall group effect was detected, a marginal group × week interaction suggested a stronger reduction in ADG in treatment pigs.

Average daily feed intake (ADFI) increased with experimental week. However, a significant group × week interaction indicated that feed intake patterns differed between groups, with treatment pigs showing a smaller increase over time.

Feed conversion ratio (FCR) showed a numerical increase across weeks, indicating a gradual reduction in feed efficiency. Nevertheless, neither the group effect nor the interaction term reached statistical significance.

When performance variables were summarized across the entire experimental period, using pen-level means, challenged pigs showed reduced productivity compared with control pigs (Table 4). Control pigs exhibited significantly higher body weight gain and ADG, while treatment pigs consumed less feed and demonstrated poorer feed conversion efficiency.

### 3.4. Physiological and Biological Responses

#### 3.4.1. Body Temperature and Respiration Rate Changes

**Figure 3:**
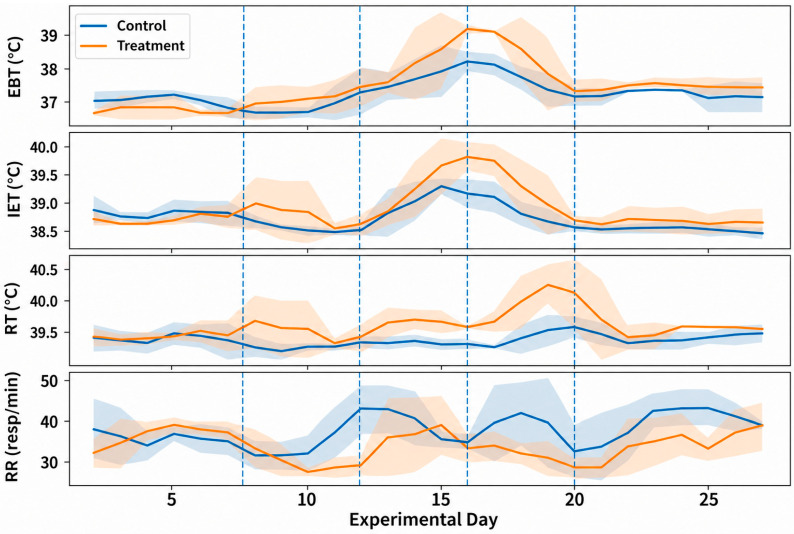
Temporal profiles of physiological responses in control and *Klebsiella pneumoniae*-challenged pigs across the 28-day experiment. Ear base temperature (EBT), inner ear temperature (IET), rectal temperature (RT), and respiration rate (RR) are shown as daily means, with shaded areas indicating ± SD. Vertical dashed lines indicate intranasal inoculation days (8, 12, 16, and 20).

Temporal changes in physiological responses are presented in Figure 3. Ear base temperature (EBT), inner ear temperature (IET), rectal temperature (RT), and respiration rate (RR) were generally comparable between groups during days 1–7. After inoculation, the challenged room showed transient increases in temperature-related and respiration-related indicators, but day-specific differences were inconsistent. These variables, therefore, provide contextual support for the acoustic response rather than strong independent physiological confirmation of disease.

#### 3.4.2. Blood Biochemical Changes

Albumin concentrations were normally distributed across all group × time combinations (*p* > 0.05) (Table A1). Mixed repeated-measures ANOVA revealed no significant main effects of group (*p* = 0.065) or time (*p* = 0.254), and no group × time interaction (*p* = 0.872). Although the group effect did not reach statistical significance, the partial eta squared indicated a moderate-to-large effect size (η^2^p = 0.46). Post hoc comparisons showed that albumin concentrations at 3WPI were numerically higher than at 0HBI; however, this difference did not remain significant after Bonferroni correction. Similarly, albumin tended to be higher in the control group than in the treatment group at 3WPI, but this difference was not statistically significant after adjustment. Therefore, albumin should be interpreted as largely stable in this moderate challenge model, not as evidence of a pronounced systemic inflammatory response.

**Figure 4:**
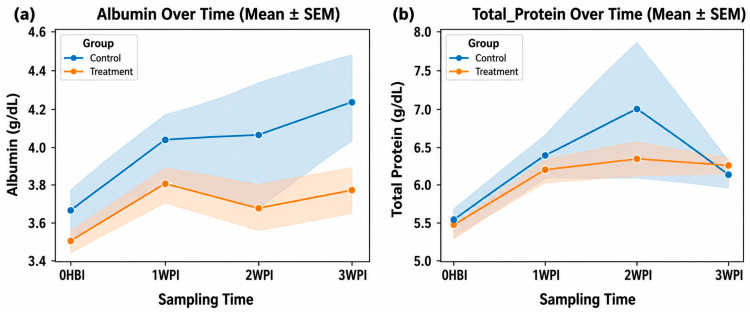
Changes in blood biochemical indicators before and after bacterial challenge. (**a**) Serum albumin concentration and (**b**) total protein concentration in control and treatment pigs at 0HBI, 1WPI, 2WPI, and 3WPI. 0HBI: 0 h before first inoculation; 1WPI: 1-week post-first inoculation; 2WPI: 2-weeks post-first inoculation; 3WPI: 3-weeks post-first inoculation.

Total protein values were normally distributed in most conditions; a slight deviation at control 0HBI was considered acceptable given the small sample size. Mauchly’s test indicated violation of sphericity; therefore, Greenhouse–Geisser correction was applied. Mixed repeated-measures ANOVA revealed no significant main effect of group (*p* = 0.437) and no significant group × time interaction (*p* = 0.748). The time effect was significant before correction (*p* = 0.019) but did not remain statistically significant after Greenhouse–Geisser adjustment (*p* = 0.057; η^2^p = 0.41). These results suggest a temporal tendency for increased total protein following inoculation, although the effect did not reach statistical significance after correction (Table A1). The trends of blood albumin and total protein in control and treatment pigs along different sampling stages are presented in Figure 4.

#### 3.4.3. Fecal Consistency

**Figure 5:**
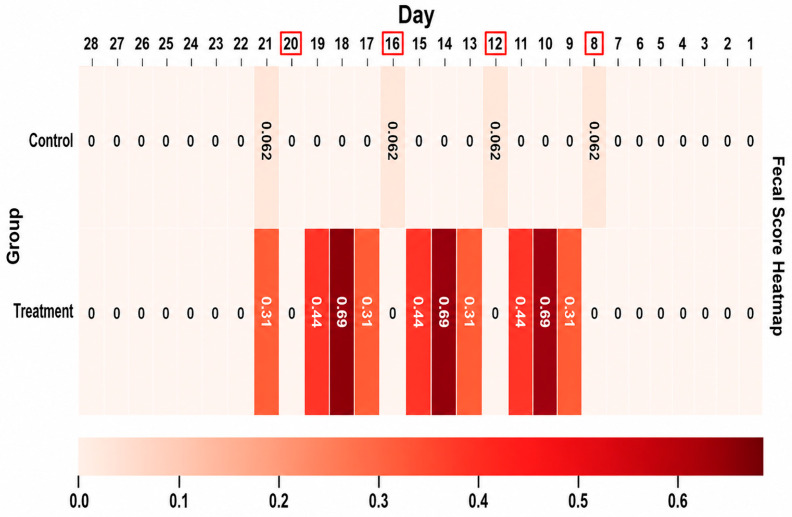
Daily fecal consistency scores in control and *Klebsiella pneumoniae*-challenged pigs during the 28-day experiment. The heatmap summarizes mean fecal score by day and group using the 0–3 scale (0 = normal, 3 = severe diarrhea), showing mild and transient post-inoculation soft feces in treatment pigs without severe diarrhea, red squares indicate inoculation days: 8, 12, 16, and 20.

Fecal consistency remained normal (score 0) in both groups from day 1 to day 8. Transient increases were subsequently observed in the treatment group on days 9–11, 13–15, 17–19, and 21 (Figure 5). In contrast, the control group showed only minimal and sporadic elevations (mean = 0.0625). The highest mean fecal scores in the treatment group occurred on days 10, 14, and 18 (mean = 0.6875; median = 0.625), corresponding predominantly to soft feces. No cases of mild or severe diarrhea were recorded. Within-group analysis suggested significant temporal variation in the treatment group (*p* < 0.001), whereas no temporal effect was detected in the control group (*p* = 0.464). Between-group comparisons at individual time points were not statistically significant (all *p* > 0.05), although fecal scores were consistently numerically higher in the treatment group during post-inoculation periods.

### 3.5. Acoustic Responses—Group-Size-Standardized Room-Level Indices

Across 5376 hourly room-level observations, acoustic activity differed between the control and challenged rooms, but all values should be interpreted as repeated room-level soundscape indices rather than independent pig-level measurements. Detailed hourly and daily model outputs are provided in Table A2, Table A3, Table A4, Table A5, Table A6 and Table A7. In the main analysis, daily aggregation showed that the challenged room had higher group-size-standardized coughing, normal-vocalization, and sneezing detections than the control room (Table 5). Coughing showed the clearest temporal divergence, with a significant group × day interaction, indicating a progressive post-inoculation increase in the challenged room. Normal vocalizations were also higher in the challenged room, whereas aggressive vocalizations remained similar between rooms.

**Figure 6:**
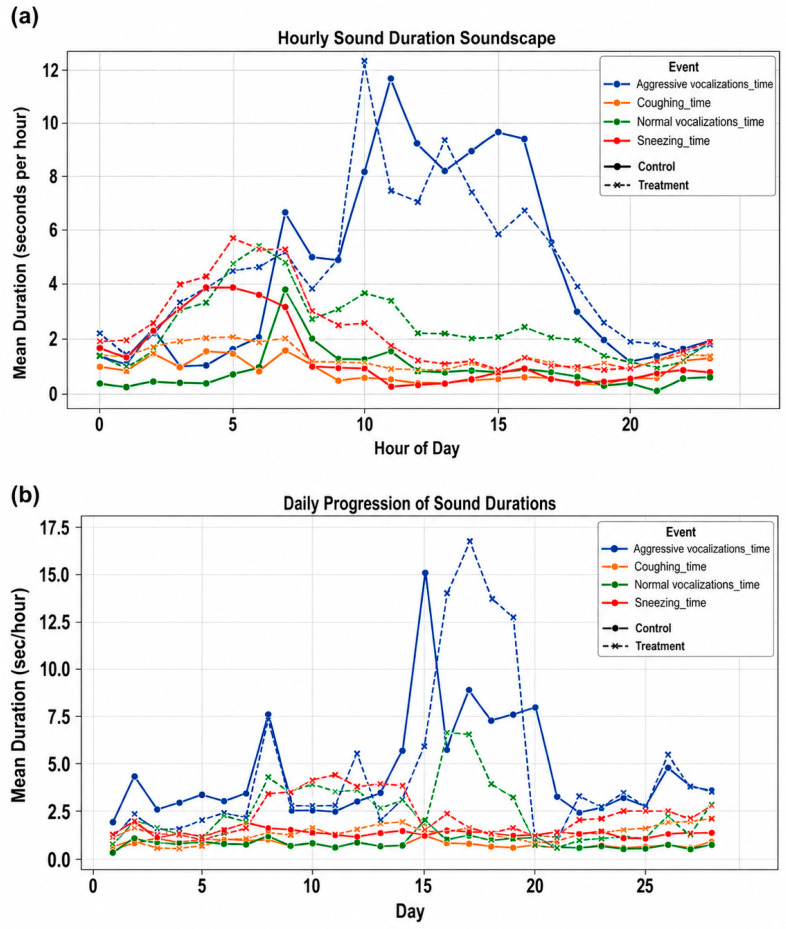
Daily and diurnal patterns of group-size-standardized room-level sound-event duration in control and treatment groups. (**a**) Mean hourly acoustic time by sound category across the 24 h cycle. (**b**) Mean daily acoustic time by sound category across the 28-day experiment. Duration indices are room-level values standardized by group size.

Daily acoustic-time indices showed the same directional pattern as detection counts. Relative to the control room, the treatment room also showed longer standardized room-level coughing (+60%), normal-vocalization (+177%), and sneezing (+63%) duration indices (Figure 6). Sensitivity analysis using merged coughing episodes and alternative probability thresholds produced the same directional post-inoculation pattern, indicating that the main room-level trend was robust, although absolute counts remained algorithm-dependent.

**Figure A3:**
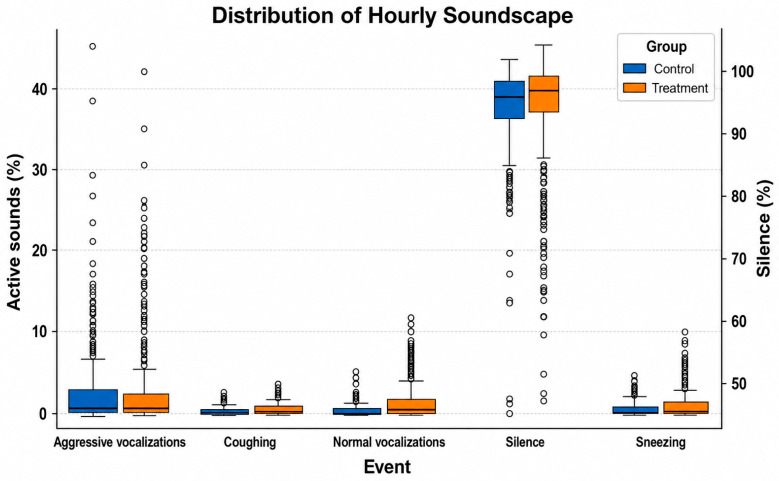
Hourly acoustic soundscape composition in control and treatment groups across the 28-day experiment. The figure shows the percentage of total recorded time occupied by silence, aggressive vocalizations, coughing, normal vocalizations, and sneezing. Percentage time occupancy was calculated at group level.

**Figure A4:**
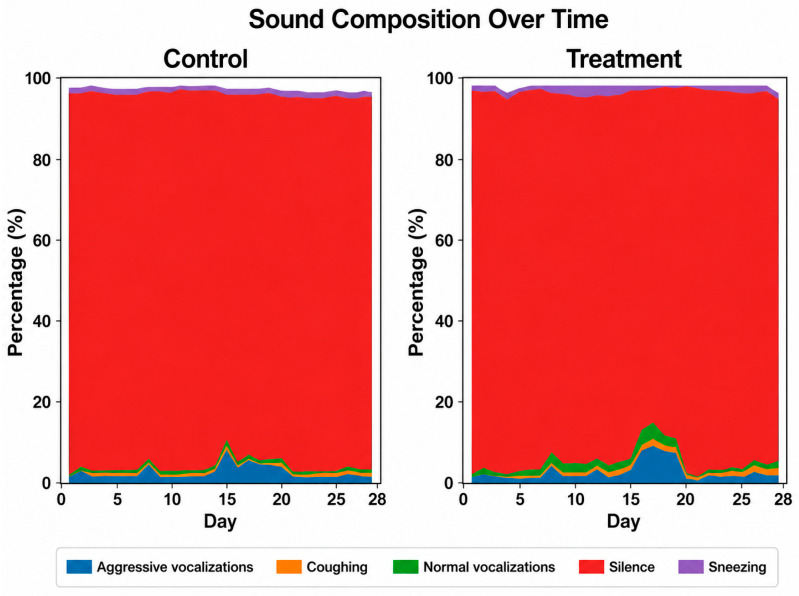
Daily soundscape composition (% time occupancy) in control and treatment groups throughout the 28-day experiment. The figure illustrates day-to-day changes in the percentage of recording time occupied by each sound class, highlighting progressive divergence in coughing occupancy and higher normal vocalization occupancy in treatment pigs. Percentage time occupancy was calculated at group level.

Soundscape composition was dominated by silence in both rooms, accounting for more than 93% of recorded time (Figure A3 and Figure A4). Among active sound classes, aggressive vocalizations occupied the largest proportion of recording time, but differed little between rooms. In contrast, coughing, normal vocalizations, and sneezing occupied a larger proportion of time in the challenged room. These findings indicate that the respiratory challenge was associated with a broader shift in room-level acoustic activity, especially increased coughing and related non-silent soundscape components, but the results remain exploratory because each condition was represented by one monitored room.

### 3.6. Acoustic and Integrated Early-Warning Responses

**Figure 7:**
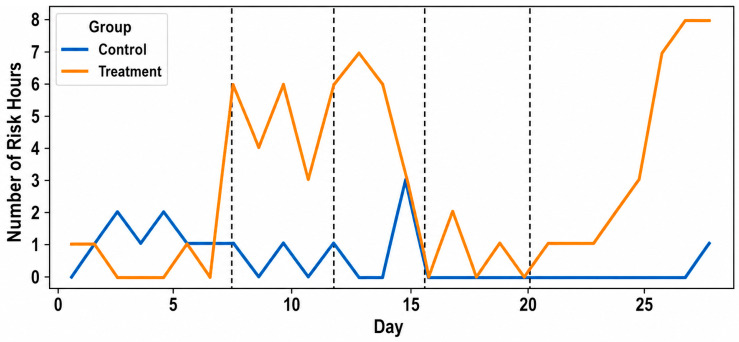
Daily occurrence of threshold-defined coughing risk windows in control and treatment rooms during the 28-day experiment. Group-specific coughing thresholds were derived from the adaptation period (days 1–7) using the equation μ + 2σ and applied to hourly room-level coughing detections. Dashed vertical lines indicate inoculation days (8, 12, 16, and 20). The treatment group showed a marked increase in daily risk windows after inoculation, with the highest daily values observed on days 27 and 28.

Continuous acoustic monitoring was used to evaluate whether baseline-derived thresholds could detect post-inoculation respiratory anomalies. Group-specific coughing thresholds were calculated from days 1–7 using mean + 2 SD; this approach was used as a transparent anomaly-detection heuristic rather than a clinically validated disease threshold. The threshold was 4.50 detections·h^−1^ in the control room and 4.23 detections·h^−1^ in the treatment room (Table 6). Application of these thresholds identified 15 coughing-risk hours in the control room and 78 in the treatment room, of which 75 occurred after day 7. The highest daily burden in the treatment room occurred on days 27 and 28, with 8 risk hours each, whereas the control room showed only sporadic exceedances, with a maximum of 3 risk hours on day 15 (Figure 7).

**Figure 8:**
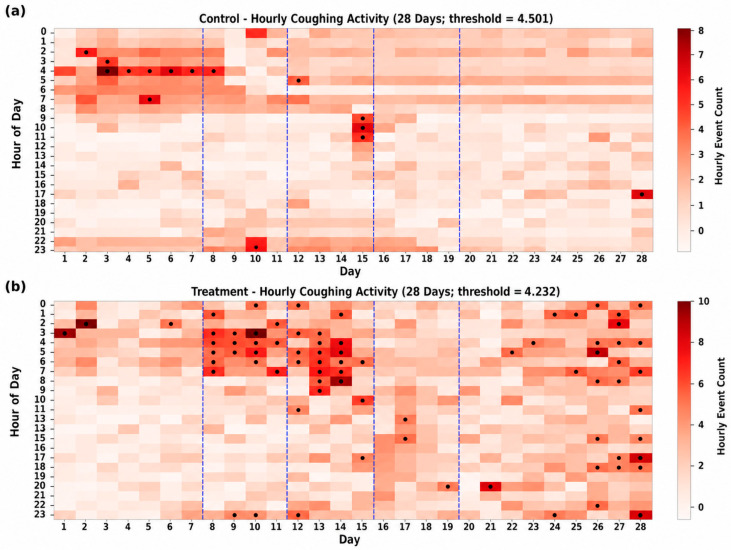
Day–hour heatmaps of hourly room-level coughing activity in (**a**) control and (**b**) treatment groups across the 28-day experiment. Black dots indicate hourly observations exceeding the respective group-specific coughing threshold derived from days 1–7, and blue dashed lines indicate inoculation days (8, 12, 16, and 20). Threshold exceedances clustered mainly during early-morning hours and were more frequent and persistent in the treatment group.

The hour-of-day distribution further suggested early-morning clustering of coughing-risk windows, especially in the treatment room. Across both rooms, the highest total frequency occurred at 04:00, followed by 05:00 and 03:00–07:00. This pattern was driven mainly by the treatment room, while the control room showed only occasional exceedances (Table A8). The day–hour heatmaps confirmed that threshold exceedances became more frequent and persistent after inoculation in the treatment room (Figure 8a,b).

### 3.7. Early Respiratory Detection Using Acoustic Signals

**Figure 9:**
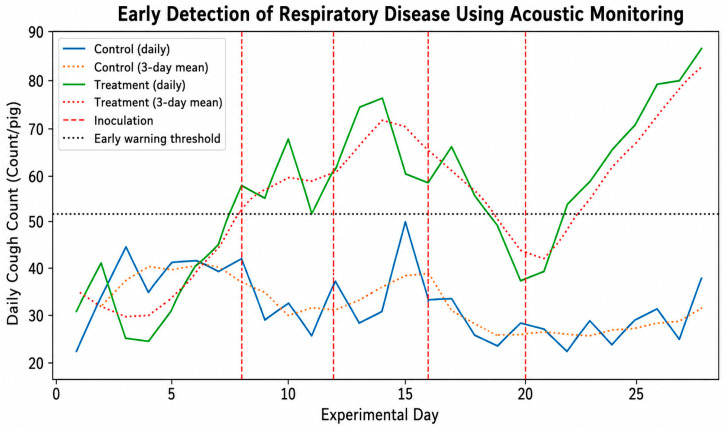
Early detection of respiratory disturbance using daily room-level coughing detections relative to the baseline-derived threshold. Daily coughing detections in control and treatment groups are shown against the threshold calculated from the pre-inoculation baseline period (days 1–7; μ + 2σ). The treatment group exceeded the threshold from day 8 onward, coinciding with the first *Klebsiella pneumoniae* inoculation, whereas the control group remained below the threshold throughout the experiment.

To evaluate whether acoustic monitoring could provide early detection of respiratory event anomalies, baseline coughing activity was first established using pre-inoculation data (days 1–7). During this baseline period, coughing activity was comparable between groups, with mean daily counts of 38.27 ± 7.05 events in the control group and 35.21 ± 8.03 events in the treatment group (Table 7). Daily coughing in the treatment room first exceeded the baseline-derived threshold on day 8, coinciding with the first inoculation, whereas the control room remained below the threshold throughout the experiment (Figure 9).

The integrated acoustic–physiological score provided a broader early-warning summary by combining coughing, sneezing, ear base temperature, inner ear temperature, rectal temperature, and respiration rate. Baseline-derived thresholds were 0.764 for the control score, 1.115 for the treatment score, and 0.704 for the treatment–control contrast score (Table 8). The treatment score and the treatment–control contrast score first exceeded the threshold on day 8, whereas the control score showed only occasional exceedances. Leave-one-day-out analysis indicated that the equal-weight integrated score discriminated post-inoculation challenge days better than coughing detections alone (AUROC 0.94 vs. 0.88; AUPRC 0.91 vs. 0.84). Alternative inverse-variance and logistic-weighted sensitivity scores produced the same first warning day and similar AUROC values (0.92–0.95).

**Figure 10:**
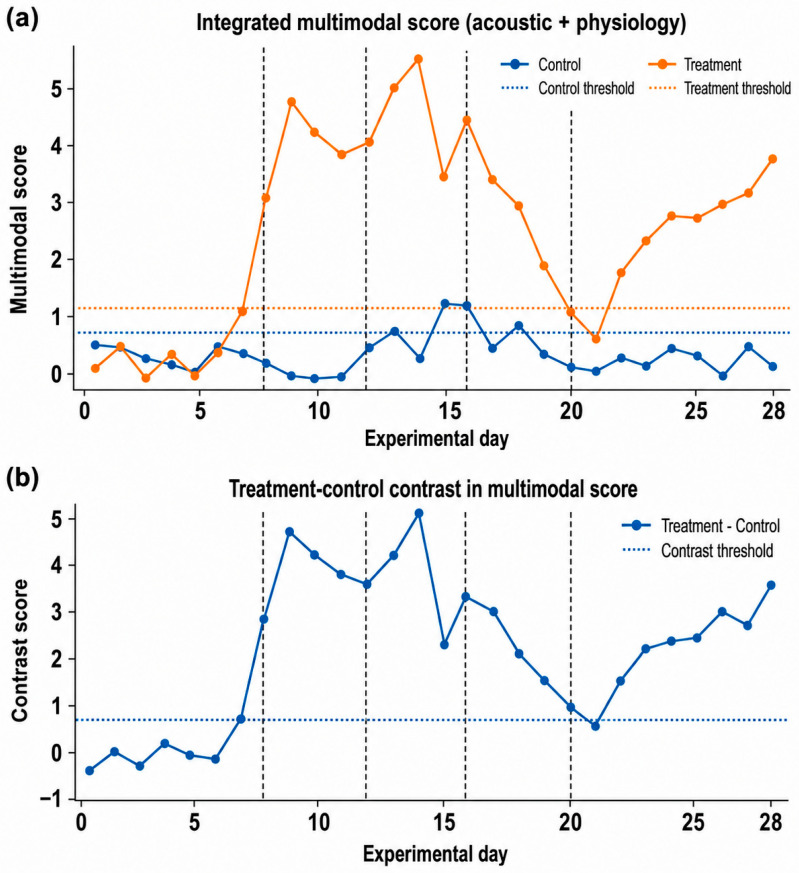
Integrated acoustic–physiological early-warning trajectories during the 28-day experiment. (**a**) Group-specific daily integrated scores for control and treatment groups with group-specific baseline-derived thresholds. (**b**) Daily treatment–control contrast score with the corresponding baseline-derived threshold. Vertical dashed lines indicate inoculation days (8, 12, 16, and 20). The integrated score fused coughing, sneezing, EBT, IET, RT, and RR. The control threshold was 0.764, the treatment threshold was 1.115, and the contrast threshold was 0.704. The first post-baseline warning day was 13 for the control score and 8 for both the treatment and contrast scores.

Following day 8, the treatment multimodal trajectory remained above threshold on most post-baseline days, and the treatment–control contrast score remained above its threshold for nearly the entire post-inoculation period (Figure 10). Overall, these findings suggest that room-level coughing activity can indicate early post-inoculation acoustic anomalies, while integration with physiological variables provides a more stable early-warning trajectory. However, these outputs should be interpreted as exploratory warning signals within a controlled two-room pilot setting and not as independent diagnostic evidence.

### 3.8. Multimodal Associations with Environmental and Physiological Variables

**Figure 11:**
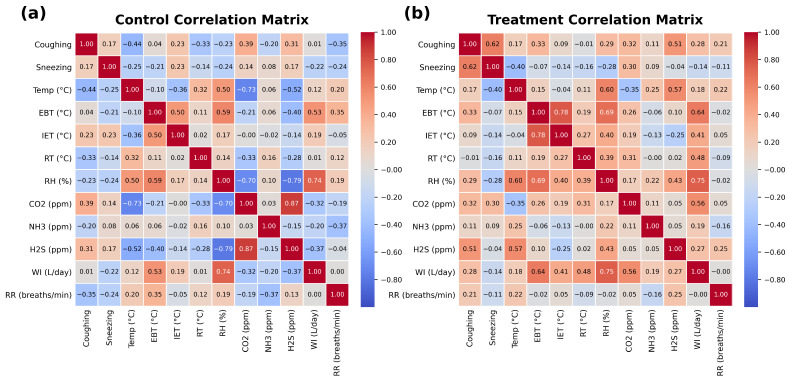
Pairwise correlation matrices of coughing, sneezing, environmental variables, and physiological measures in (**a**) control and (**b**) treatment groups during the 28-day experiment. The heatmaps show Pearson correlation coefficients among coughing, sneezing, room temperature (Temp), ear base temperature (EBT), inner ear temperature (IET), rectal temperature (RT), relative humidity (RH), CO_2_, NH_3_, H_2_S, WI, and respiration rate (RR). Stronger and more coherent correlations with coughing were observed in the treatment group than in the control group.

The control and treatment rooms showed different microclimatic profiles, but most measured values remained within broadly acceptable operational ranges for pig housing (Table A9). Pairwise correlation patterns differed noticeably between the control and treatment groups (Figure 11). In the control group, coughing showed relatively weak and inconsistent associations with the monitored variables. The correlation between coughing and sneezing was low (r = 0.17), and coughing showed mixed relationships with environmental and physiological measures, including negative correlations with room temperature (r = −0.44), rectal temperature (r = −0.33), and respiration rate (r = −0.35), and modest positive correlations with CO_2_ (r = 0.39) and H_2_S (r = 0.31). Overall, the control heatmap suggested only limited coupling between coughing activity and the surrounding physiological or environmental context.

In contrast, the treatment group showed a more coherent multimodal pattern. Coughing was strongly positively correlated with sneezing (r = 0.62) and moderately positively correlated with H_2_S (r = 0.51), ear base temperature (r = 0.33), CO_2_ (r = 0.32), relative humidity (r = 0.29), and respiration rate (r = 0.21). These associations indicate that coughing activity in challenged pigs was more closely linked with concurrent respiratory, physiological, and environmental changes than in the control group. However, because environmental measurements were recorded at the room level, and each group occupied one room, these associations describe contextual co-variation rather than causal environmental effects.

Exploratory multivariable regression analysis supported this group difference. In the control group, the regression model was not statistically significant (R^2^ = 0.294, *p* = 0.149), and none of the environmental or physiological variables were significantly associated with coughing frequency. In contrast, the model for treatment pigs was statistically significant (R^2^ = 0.658, *p* < 0.001) and explained a substantial proportion of the variation in coughing activity. CO_2_ concentration (β = 0.204, *p* = 0.005) and experimental day (β = 2.003, *p* < 0.001) were positively associated with coughing frequency, indicating increasing respiratory activity as the experiment progressed. Relative humidity showed a marginal negative association (β = −4.16, *p* = 0.053), whereas NH_3_ concentration and rectal temperature were not significant predictors (Table A10). These results are hypothesis-generating because they are based on repeated observations from the same two rooms.

## 4. Discussion

### 4.1. Validation of the Infection Model

The challenge data support respiratory colonization, as indicated by significantly higher presumptive nasal bacterial counts in the treatment group. Nasal bacterial load is widely used as an indicator of respiratory colonization in pigs because the nasal cavity represents a primary reservoir for respiratory pathogens and reflects infection dynamics within the upper respiratory tract [22,23,24]. In contrast, fecal bacterial shedding remained low and did not differ between groups, suggesting that the infection remained largely localized to the respiratory tract. Similar patterns have been reported in experimental respiratory infections in pigs, where nasal shedding occurs more frequently than fecal shedding due to pathogen tropism for respiratory tissues [27,28]. The increase in nasal bacterial load during the post-inoculation period and the higher cumulative exposure (AUC) further suggested sustained colonization rather than transient contamination.

Although bacterial counts gradually declined over time, detectable colonization persisted throughout the experiment, suggesting partial host immune adaptation without complete pathogen clearance. Such persistence is commonly observed during respiratory infections in pigs, where immune responses can reduce pathogen load without fully eliminating colonization [22,23]. The absence of detectable bacteria prior to inoculation further suggests that colonization resulted from the experimental challenge rather than pre-existing infection. Because this study used a single strain and one intended dose, the results should not be generalized to all *K. pneumoniae* strains, doses, or field infection scenarios.

The co-occurrence of higher nasal burden, more coughing, and poorer growth supports the interpretation that the challenge affected respiratory health and performance [17,29,30,31,32,33], but the AST component itself classified sounds rather than diagnosing infection.

### 4.2. Growth Performance and Physiological Responses

Challenged pigs showed lower BWG, lower ADG, and poorer feed-use outcomes than controls, which is consistent with reported production costs of respiratory challenge [5,32,34,35,36]. However, these performance findings should be interpreted as pen-level summaries nested within one room per condition, not as fully replicated treatment effects.

Physiological indicators suggested a mild to moderate contextual response, but they were less consistent than nasal bacterial recovery and coughing acoustic activity. Serum albumin remained largely stable. The stability of albumin levels suggests that the infection remained largely localized within the respiratory tract, as it decreases in its concentration with systemic inflammation or severe infection [37,38]. In contrast, the gradual increase in total serum protein may reflect activation of the humoral immune response, as infections often stimulate the production of immunoglobulins and other immune-related proteins that contribute to increased globulin fractions in serum [39,40,41]. However, these variables support interpretation as contextual physiological layers rather than strong biochemical confirmation of systemic disease.

Fecal consistency scores indicated mild and transient soft feces after inoculation without severe diarrhea. This pattern is consistent with a challenge that primarily affected the respiratory tract rather than causing broad gastrointestinal disease [9].

### 4.3. Acoustic Indicators of Respiratory Infection

Recent advances in machine learning have enabled automated sound-classification systems capable of detecting pig coughs and other acoustic events under farm conditions [17,42,43,44,45]. Among the evaluated sound events, coughing emerged as the most informative acoustic biomarker, showing a clear temporal increase during the course of infection. Previous studies have demonstrated that cough frequency is strongly associated with respiratory infections in pigs [16,46]. Sneezing was also elevated in infected pigs, suggesting irritation of the upper respiratory tract [47,48]. In contrast, aggressive vocalizations were not significantly affected by infection status, indicating that social interactions among pigs were not substantially altered under the experimental conditions. The increase in normal vocalizations observed in treatment pigs may reflect behavioral responses to discomfort or respiratory distress, as animal vocalizations are known to change in response to stress or illness [49,50]. However, disease inference came from the combined context of inoculation timing, presumptive nasal *K. pneumoniae* recovery, growth response, and physiological trends, not from the AST output alone.

### 4.4. Temporal Dynamics of Respiratory Symptoms

Time-resolved acoustic monitoring revealed episodic increases in coughing detections after inoculation. These peaks should be interpreted as threshold-defined surveillance windows rather than clinically validated risk periods. Similar episodic patterns have been reported in pig production systems, where coughing frequency increases during periods of active infection or disease outbreaks [16,51,52].

Coughing and sneezing were positively associated, suggesting simultaneous irritation of both upper and lower respiratory tract regions. However, coughing appeared to be the more sensitive indicator of respiratory compromise, consistent with previous reports linking cough frequency with lung lesions and pneumonia in pigs [16].

The strongest concentration of coughing threshold exceedances occurred between 03:00 and 07:00, with a peak around 04:00, is in line with previous report stating circadian rhythms influence pig behavior, activity patterns, and physiological processes, with daily peaks in activity typically occurring in the early morning and evening [53], but feeding, lighting, human activity, room conditions, and post-inoculation progression may also influence timing. Therefore, this manuscript avoids claiming a proven circadian cough mechanism.

### 4.5. Environmental Influences on Coughing Activity

Environmental conditions may also contribute to temporal fluctuations in respiratory symptoms. Air quality is a critical determinant of respiratory health in pig production systems. Elevated concentrations of ammonia, carbon dioxide, dust, and humidity have been associated with increased coughing frequency and higher susceptibility to respiratory disease outbreaks [7,54,55,56]. Poor ventilation and elevated airborne pollutants can exacerbate respiratory irritation and increase disease susceptibility [8,55,57]. The side-by-side correlation matrices indicate that environmental associations were more evident in the treatment group than in the control group. However, because environmental measurements were recorded at the room level, and each group occupied a separate room, these associations should be interpreted as contextual co-variation rather than direct causal effects.

### 4.6. Early Acoustic Detection of Respiratory Disturbance

Baseline-derived daily coughing thresholds were exceeded in the treatment group from day 8, the same day as the first inoculation, supporting acoustic monitoring as a possible early-anomaly signal [58,59,60]. The integrated acoustic–physiological score strengthened the warning trajectory, but the threshold rule remains heuristic and requires external validation before on-farm deployment.

## 5. Limitations

This study has several important limitations. First, acoustic monitoring was performed with one microphone per room, so detections represented room-level soundscapes and could not be assigned to pens or individual pigs. Second, each experimental condition was represented by a single room; therefore, room effects, treatment effects, airflow, room acoustics, microclimate, and social dynamics cannot be fully separated for acoustic, environmental, or integrated-score analyses. Third, the AST model was trained and internally validated within the same facility, which limits external generalizability even after leakage-aware and temporal validation. Fourth, the integrated score used equal weighting as a transparent pilot rule; although sensitivity analyses were positive, the score has not been validated prospectively. The integrated score fused only daily acoustic and physiological variables; environmental, bacteriological, and blood data were retained as contextual or low-frequency validation layers rather than fully fused inputs. Fifth, the challenge used one *K. pneumoniae* strain and one intended dose under controlled conditions, so field infections, co-infections, and different pathogen loads may produce different acoustic responses. Multi-room, multi-farm, and prospective validation are required before broader deployment.

## 6. Conclusions

In this two-room pilot study, room-level AST-based acoustic monitoring provided a feasible summary of coughing and related soundscape changes after an experimental *K. pneumoniae* challenge. Challenged pigs showed presumptive nasal bacterial recovery, lower growth performance, more threshold-defined coughing risk windows, and an integrated acoustic–physiological score that diverged from controls from day 8 onward. These findings support the concept of combining room-level sound monitoring with simple physiological measurements as an early-warning framework under controlled challenge conditions. However, the approach is not a stand-alone diagnostic system, and replicated multi-room and multi-farm validation is needed before broader on-farm inference or deployment.

## Figures and Tables

**Table 1:** Classification performance of the fine-tuned Audio Spectrogram Transformer (AST) for pig sound recognition and validation against manual hourly annotations.

Sound Class	Precision	Recall	F1-Score	Pearson r	RMSE	MAE
Coughing event	0.94	0.95	0.94	0.99	6.17	6.00
Sneezing	0.98	0.88	0.93	0.99	8.38	7.96
Aggressive vocalizations	0.88	0.97	0.92	0.98	17.28	13.13
Normal vocalizations	0.96	0.96	0.96	0.99	7.76	7.29
Silence	0.98	0.99	0.99	–	–	–
Test accuracy			0.949	–	–	–
Test macro-F1			0.947	–	–	–
Five-fold group-blocked macro-F1	–	–	0.928 ± 0.019	–	–	–
24 h temporal deployment macro-F1	–	–	0.914	–	–	–
Threshold/window sensitivity		–	r = 0.93–0.98	–	–	–

Precision, recall, and F1-score were calculated on the independent test set (20% of labeled audio segments). Pearson’s r, RMSE, and MAE were calculated by comparing automated hourly event counts with manual annotations from 24 h validation recordings. A dash indicates not applicable. RMSE: root mean square error; MAE: mean absolute error.

**Table 2:** Presumptive *Klebsiella/K. pneumoniae* loads in nasal and fecal samples from control and inoculated pigs at baseline and weekly post-inoculation sampling points.

Sample	Time	Control (Mean ± SD)	Treatment (Mean ± SD)	Welch *p*-Value	Mann–Whitney *p*-Value
Nasal swab	0HBI	0.00 ± 0.00	0.00 ± 0.00	–	1.000
Nasal swab	1WPI	0.67 ± 1.35	4.03 ± 0.27	0.0137	0.0265
Nasal swab	2WPI	1.35 ± 1.56	3.57 ± 0.58	0.0587	0.0530
Nasal swab	3WPI	0.67 ± 1.35	3.00 ± 0.25	0.0388	0.0360
Fecal	0HBI	0.00 ± 0.00	0.00 ± 0.00	–	1.000
Fecal	1WPI	0.00 ± 0.00	0.67 ± 1.35	0.3910	0.4533
Fecal	2WPI	0.00 ± 0.00	1.47 ± 1.71	0.1838	0.1859
Fecal	3WPI	0.67 ± 1.35	1.91 ± 2.20	0.3843	0.4084

Values are mean ± SD log_10_ CFU per swab for nasal samples or per gram of feces for fecal samples. Colonies were enumerated as presumptive *Klebsiella/K. pneumoniae* based on expected purple–magenta mucoid morphology on Klebsiella ChromoSelect selective agar. No molecular, MALDI-TOF, sequencing, or biochemical confirmation was performed for any recovered colonies; therefore, values represent presumptive culture-based recovery rather than definitive species-level confirmation. 0HBI: 0 h before first inoculation; 1WPI: 1-week post-first inoculation; 2WPI: 2-weeks post-first inoculation; 3WPI: 3-weeks post-first inoculation. Welch’s *t*-test and Mann–Whitney U test were used for between-group comparisons at each sampling point.

**Table 3:** Linear mixed-effects model estimates for weekly growth performance responses in control and *Klebsiella pneumoniae*-challenged pigs.

Variable	Effect	Estimate	SE	z-Value	*p*-Value
BW	Group	1.612	1.102	1.463	0.143
	Week	7.250	0.318	22.799	<0.001
	Group × Week	−2.984	0.450	−6.635	<0.001
ADG	Group	−0.001	0.228	−0.005	0.996
	Week	−0.124	0.059	−2.105	0.035
	Group × Week	−0.157	0.083	−1.886	0.059
ADFI	Group	0.268	0.316	0.848	0.397
	Week	0.232	0.082	2.843	0.004
	Group × Week	−0.232	0.115	−2.011	0.044
FCR	Group	0.541	1.070	0.506	0.613
	Week	0.465	0.276	1.685	0.092
	Group × Week	0.579	0.391	1.483	0.138

Estimates are fixed-effect coefficients from the fitted linear mixed-effects models. BW: body weight; ADG: average daily gain; ADFI: average daily feed intake; FCR: feed conversion ratio; SE: standard error.

**Table 4:** Overall growth performance of control and *Klebsiella pneumoniae*-challenged pigs during the 28-day experiment.

Parameter	Control Pen (Mean ± SD)	Challenged Pen (Mean ± SD)	Adjusted Difference (95% CI)	Descriptive *p*-Value
Initial BW (kg)	52.74 ± 0.40	52.80 ± 0.38	+0.06 (−0.58 to 0.70)	0.836
Overall BWG (kg)	29.63 ± 1.95	18.59 ± 1.33	−11.04 (−13.20 to −8.88)	<0.001
Overall ADG (kg/day)	1.06 ± 0.07	0.67 ± 0.05	−0.39 (−0.47 to −0.31)	<0.001
Overall ADFI (kg/day)	2.58 ± 0.16	2.27 ± 0.14	−0.31 (−0.51 to −0.10)	0.028
Overall FCR	2.44 ± 0.11	3.42 ± 0.15	+0.98 (+0.70 to +1.26)	<0.001

Values are pen-level mean ± SD for the overall experimental period (n = 4 pens per room). Adjusted differences are treatment minus control with bootstrap 95% confidence intervals. *p*-values are descriptive because pens were nested within one room per condition.

**Table 5:** Summary of daily room-level acoustic detection, acoustic-time, and soundscape occupancy indices in control and challenged rooms.

Sound Event	Daily Detections: Control	Daily Detections: Challenged	Daily Acoustic Time: Control	Daily Acoustic Time: Challenged	Daily Occupancy: Control (%)	Daily Occupancy: Challenged (%)
Aggressive vocalizations	181.49 ± 114.79	185.77 ± 173.65	109.25 ± 68.92	111.46 ± 104.19	2.53 ± 1.60	2.58 ± 2.41
Coughing	36.80 ± 7.27	54.84 ± 16.49	22.50 ± 4.25	32.90 ± 9.89	0.52 ± 0.10	0.76 ± 0.23
Normal vocalizations	36.00 ± 11.76	100.99 ± 63.77	21.89 ± 7.05	60.59 ± 38.26	0.51 ± 0.16	1.40 ± 0.89
Sneezing	56.67 ± 8.02	92.72 ± 42.27	34.00 ± 4.81	55.63 ± 25.36	0.79 ± 0.11	1.29 ± 0.59
Silence	—	—	—	—	94.67 ± 1.74	93.77 ± 3.24

Values are mean ± SD. Daily detections are group-size-standardized room-level detections·day^−1^, acoustic time is group-size-standardized s·day^−1^, and occupancy is the percentage of daily recorded time. These values represent room-level acoustic indices, not individual-pig measurements. Detailed hourly models, daily model *p*-values, group × day effects, and sensitivity analyses are provided in Table A2, Table A3, Table A4, Table A5, Table A6 and Table A7.

**Table 6:** Summary of baseline-derived coughing thresholds, daily respiratory event frequency, and threshold-defined coughing risk windows during the 28-day experiment.

Parameter	Control	Treatment
Baseline mean hourly cough detections (days 1–7; detections·h^−1^)	1.59	1.47
Baseline SD of hourly cough count (days 1–7)	1.45	1.38
Group-specific coughing threshold (μ + 2σ; detections·h^−1^)	4.50	4.23
Mean daily cough detections (detections·day^−1^)	36.80	54.84
Mean daily sneeze detections (detections·day^−1^)	56.67	92.72
Total coughing risk hours	15	78
Maximum coughing risk hours in a single day	3	8

Baseline mean, SD, and threshold were calculated from hourly room-level coughing detections during days 1–7 separately for each group. Daily cough and sneeze counts are room-level detections·day^−1^ standardized by group size. Coughing risk hours indicate hourly observations in which coughing exceeded the respective group-specific threshold.

**Table 7:** Pre- versus post-inoculation comparison of daily cough and sneeze frequencies in control and challenged pigs.

Group	Variable	Pre-Mean	Post Mean	t-Statistic	*p*-Value
Control	Daily Cough	38.27	31.94	2.097	0.063
Control	Daily Sneeze	60.71	55.43	1.070	0.321
Treatment	Daily Cough	35.21	61.38	−6.311	<0.001
Treatment	Daily Sneeze	54.81	105.35	−5.229	<0.001

Pre-inoculation period = days 1–7; post-inoculation period = days 8–28. Values are mean daily room-level detections standardized by group size. t-statistics and *p*-values compare the pre- and post-inoculation periods within each group.

**Table 8:** Baseline-derived thresholds and warning performance of the integrated multimodal score.

Analysis	Baseline Mean	Baseline SD	Threshold (μ + 2σ)	First Post-Baseline Warning Day	Warning Days During Days 8–28
Control multimodal score	0.389	0.187	0.764	13	4/21
Treatment multimodal score	0.385	0.365	1.115	8	19/21
Treatment–control contrast score	−0.004	0.354	0.704	8	20/21

The integrated multimodal score was calculated from daily acoustic and physiological variables. Acoustic inputs included coughing and sneezing, and physiological inputs included EBT, IET, RT, and RR. Baseline statistics were calculated from days 1–7.

## Data Availability

The raw data supporting the conclusions of this article will be made available by the authors on request. The datasets used to generate the reported results were submitted during the journal submission process. However, public deposition of the complete raw dataset—particularly the continuous audio recordings—is restricted because the recordings were collected from a university-operated pig farm under specific access agreements, and open redistribution may raise concerns related to farm privacy, biosecurity, and controlled-access research environments. Processed datasets supporting the findings of this study, including labeled sound events, model performance metrics, aggregated event counts, and analysis scripts, are available from the corresponding authors on reasonable request.

## Abbreviations

The following abbreviations are used in this manuscript:

AST	Audio Spectrogram Transformer
AI	Artificial Intelligence
ADG	Average Daily Gain
CFU	Colony Forming Unit
BWG	Body Weight Gain
ADFI	Average Daily Feed Intake
FCR	Feed Conversion Ratio
PCA	Principal Component Analysis
RMSE	Root Mean Square Error
MAE	Mean Absolute Error
ROC	Receiver Operating Characteristic
AUC	Area Under the Curve
BW	Body Weight
EBT	Ear Base Temperature
IET	Inner Ear Temperature
RT	Rectal Temperature
RR	Respiration Rate

## Appendix A

**Table A1:** Mixed repeated-measures ANOVA of pen-level serum albumin and total protein concentrations before and after bacterial inoculation.

Variable	Effect	df1	df2	F-Statistic	*p*-Value	η^2^p
Albumin	Group	1	6	5.11	0.065	0.460
	Time	3	18	1.48	0.254	0.198
	Group × Time	3	18	0.23	0.872	0.037
Total Protein	Group	1	6	0.69	0.437	0.104
	Time ^†^	3	18	4.25	0.057 ^‡^	0.415
	Group × Time	3	18	0.41	0.748	0.064

Values were averaged within pen before analysis; pen (n = 4 per group) was the experimental unit. η^2^p, partial eta squared. ^†^ Greenhouse–Geisser correction was applied because of violation of sphericity; ^‡^ corrected *p*-value.

**Table A2:** Hourly group-size-standardized room-level acoustic detection rates and mixed-effects model results in control and challenged groups.

Event	Control (Mean ± SD, Detections·h^−1^)	Treatment (Mean ± SD, Detections·h^−1^)	Group Effect (β)	Group *p*-Value	Hour *p*-Value	Day *p*-Value	Group × Day *p*-Value
Aggressive vocalizations	7.56 ± 12.11	7.74 ± 13.72	−1.46	0.277	0.321	0.516	0.163
Coughing	1.40 ± 1.11	2.28 ± 1.66	−0.12	0.412	<0.001	0.095	<0.001
Normal vocalizations	1.50 ± 1.90	4.21 ± 5.54	2.81	<0.001	<0.001	0.807	0.787
Sneezing	2.36 ± 2.32	3.86 ± 4.33	1.03	0.003	<0.001	0.553	0.119

Values are mean ± SD room-level detections·h^−1^ standardized by group size. These counts do not represent individual-pig measurements. β denotes the fitted group coefficient (treatment relative to control). *p*-values correspond to the tested effects of group, hour, day, and group × day.

**Table A3:** Descriptive statistics of hourly group-size-standardized room-level acoustic-time indices in control and challenged groups.

Event	Control (Mean ± SD, s·h^−1^)	Treatment (Mean ± SD, s·h^−1^)	Group Effect (β)	Group *p*-Value	Hour *p*-Value	Day *p*-Value	Group × Day (*p*)
Aggressive vocalizations	4.55 ± 7.26	4.64 ± 8.23	−0.87	0.314	0.369	0.161	0.203
Coughing	0.85 ± 0.67	1.37 ± 1.00	−0.08	0.371	<0.001	0.025	<0.001
Normal vocalizations	0.91 ± 1.14	2.52 ± 3.32	1.68	<0.001	<0.001	0.660	0.766
Sneezing	1.42 ± 1.39	2.32 ± 2.60	0.62	0.004	<0.001	0.354	0.131

Values are mean ± SD s·h^−1^ standardized by group size. These values are room-level acoustic-time indices rather than individual-pig measurements. β represents the estimated treatment effect relative to the control group. *p*-values correspond to the tested effects of group, hour, day, and group × day interaction in the linear model. Linear regression models were applied to hourly duration data due to convergence limitations of mixed-effects models.

**Table A4:** Daily group-size-standardized room-level acoustic detection rates in control and challenged groups: mixed-effects model results.

Sound Event	Control (Mean ± SD, Detections·day^−1^)	Treatment (Mean ± SD, Detections·day^−1^)	Group Effect (β)	Group *p*-Value	Day *p*-Value	Group × Day *p*-Value	Cohen’s d
Aggressive vocalizations	181.49 ± 114.79	185.77 ± 173.65	−34.96	0.540	0.554	0.431	0.03
Coughing	36.80 ± 7.27	54.84 ± 16.49	−5.44	0.282	0.191	< 0.001	1.42
Normal vocalizations	36.00 ± 11.76	100.99 ± 63.77	67.46	0.005	0.829	0.907	1.42
Sneezing	56.67 ± 8.02	92.72 ± 42.27	24.63	0.145	0.631	0.439	1.18

Values are mean ± SD room-level detections·day^−1^ standardized by group size and derived from daily aggregation of hourly detection counts. These values do not represent individual-pig measurements. *p*-values were obtained from linear mixed-effects models including group, day, and group × day. Cohen’s d represents the standardized between-group effect size.

**Table A5:** Daily group-size-standardized room-level acoustic-time indices in control and challenged groups.

Event	Control (Mean ± SD, s·day^−1^)	Treatment (Mean ± SD, s·day^−1^)	Group Effect (β)	Group *p*-Value	Day *p*-Value	Group × Day *p*-Value	Effect Size (Cohen’s d)
Aggressive vocalizations	109.25 ± 68.92	111.46 ± 104.19	−0.38	0.108	0.388	0.282	0.03
Coughing	22.50 ± 4.25	32.90 ± 9.89	−0.11	0.280	0.226	<0.001	1.37
Normal vocalizations	21.89 ± 7.05	60.59 ± 38.26	0.87	<0.001	0.594	0.880	1.41
Sneezing	34.00 ± 4.81	55.63 ± 25.36	0.17	0.321	0.467	0.138	1.18

Values are mean ± SD s·day^−1^ standardized by group size and derived from daily aggregation of hourly acoustic time. These values are room-level duration indices rather than individual-pig measurements. β denotes the estimated treatment effect relative to the control group from the mixed-effects model. Cohen’s d represents standardized between-group effect size.

**Table A6:** Hourly soundscape composition (% time occupancy at group level) and statistical model results in control and challenged pigs.

Sound Event	Control Mean ± SD (%)	Treatment Mean ± SD (%)	Group *p*-Value	Hour *p*-Value	Day *p*-Value	Group × Day *p*-Value	Cohen’s d
Aggressive vocalizations	2.53 ± 4.04	2.58 ± 4.57	0.314	0.369	0.160	0.203	0.01
Coughing	0.47 ± 0.37	0.76 ± 0.55	0.371	<0.001	0.024	<0.001	0.61
Normal vocalizations	0.51 ± 0.64	1.40 ± 1.85	<0.001	<0.001	0.657	0.769	0.65
Sneezing	0.79 ± 0.77	1.29 ± 1.44	0.004	<0.001	0.354	0.131	0.43
Silence	93.59 ± 6.04	93.96 ± 7.23	0.047	<0.001	0.322	0.087	0.05

Values are mean ± SD percentage of recorded time occupied by each sound class at the group level. Percentage occupancy values are group-level time budgets and are not normalized per pig. *p*-values correspond to the tested effects of group, hour, day, and group × day. Cohen’s d represents the standardized between-group effect size.

**Table A7:** Daily soundscape composition (% time occupancy at group level) across the 28-day experiment in control and challenged pigs.

Sound Event	Control Mean ± SD (%)	Treatment Mean ± SD (%)	Group *p*-Value	Day *p*-Value	Group × Day *p*-Value	Cohen’s d
Aggressive vocalizations	2.53 ± 1.60	2.58 ± 2.41	0.540	0.546	0.439	−0.02
Coughing	0.52 ± 0.10	0.76 ± 0.23	0.292	0.268	<0.001	−1.37
Normal vocalizations	0.51 ± 0.16	1.40 ± 0.89	0.005	0.847	0.893	−1.41
Sneezing	0.79 ± 0.11	1.29 ± 0.59	0.144	0.631	0.439	−1.19
Silence	94.67 ± 1.74	93.77 ± 3.24	0.594	0.307	0.794	0.34

Values are mean ± SD percentage of daily recorded time occupied by each sound class at the group level. Percentage occupancy values are group-level time budgets and are not normalized per pig. *p*-values correspond to the tested effects of group, day, and group × day. Cohen’s d represents the standardized between-group effect size.

**Table A8:** Group-specific hour-of-day distribution of threshold-defined respiratory risk windows across the experimental period.

Hour of Day	Control Risk Occurrence	Treatment Risk Occurrence	Total Risk Occurrence
00:00	0	4	4
01:00	0	5	5
02:00	1	4	5
03:00	1	6	7
04:00	6	10	16
05:00	1	8	9
06:00	0	7	7
07:00	1	6	7
08:00	0	4	4
09:00	1	1	2
10:00	1	1	2
11:00	1	2	3
12:00	0	1	1
14:00	0	3	3
16:00	0	3	3
17:00	1	3	4
19:00	0	2	2
21:00	0	1	1
22:00	0	2	2
23:00	1	5	6

Risk occurrence indicates the number of hourly observations in which coughing exceeded the respective group-specific threshold derived from the first 7 days of the experiment. Hours with zero risk occurrence in both groups (13:00, 15:00, 18:00, and 20:00) are omitted for brevity.

**Table A9:** Summary of daily room-level environmental conditions in control and treatment rooms during the 28-day experiment.

Variable	Control (Mean ± SD)	Treatment (Mean ± SD)	Mean diff (T − C)	Effect Size (dz)	Test	*p*-Value
CO_2_ (ppm)	624.39 ± 33.30	701.99 ± 33.86	77.60	1.81	Wilcoxon	<0.001
H_2_S (ppm)	6.45 ± 0.21	5.47 ± 0.28	−0.98	−2.57	Paired *t*-test	<0.001
NH_3_ (ppm)	0.62 ± 0.29	0.48 ± 0.24	−0.15	−0.41	Paired *t*-test	0.037
Relative humidity (%)	52.75 ± 1.55	54.82 ± 1.57	2.07	4.62	Wilcoxon	<0.001
Temperature (°C)	26.05 ± 1.01	25.96 ± 1.17	−0.09	−0.16	Wilcoxon	0.186

Values are mean ± SD of daily room-level measurements recorded over the 28-day experimental period. Mean difference was calculated as treatment minus control (T − C). Effect size (dz) was calculated from paired day-wise differences. Paired *t*-tests or Wilcoxon signed-rank tests were applied as appropriate according to the distribution of day-wise paired differences. CO_2_, carbon dioxide; H_2_S, hydrogen sulfide; NH_3_, ammonia.

**Table A10:** Group-specific multivariable linear regression models of environmental, physiological, and temporal predictors of coughing frequency.

Predictor	Control β	SE	*p*-Value	Treatment β	SE	*p*-Value
Intercept	215.44	409.98	0.604	610.08	290.40	0.047
NH_3_	−4.08	4.62	0.387	−7.14	9.09	0.440
CO_2_	0.092	0.059	0.136	0.204	0.065	0.005
Relative humidity (RH)	1.52	1.46	0.309	−4.16	2.04	0.053
Rectal temperature (RT)	−7.82	9.40	0.414	−12.49	7.94	0.130
Experimental day	−0.294	0.216	0.188	2.003	0.370	<0.001
Model R^2^	0.294			0.658		
Model *p*-value	0.149			<0.001		

Separate multivariable linear regression models were fitted for control and treatment groups. β, unstandardized regression coefficient; SE, standard error; NH_3_, ammonia; CO_2_, carbon dioxide; RH, relative humidity; RT, rectal temperature.
